# Heterogeneous feature-aware Transformer-CNN coupling network for person re-identification

**DOI:** 10.7717/peerj-cs.1098

**Published:** 2022-09-27

**Authors:** Yanchao Li, Guoyun Lian, Wenyu Zhang, Guanglin Ma, Jin Ren, Jinfeng Yang

**Affiliations:** 1 School of Computer Science and Software Engineering, University of Science and Technology Liaoning, Anshan, Liaoning, China; 2 Institute of Applied Artificial Intelligence of the Guangdong-Hong Kong-Macao Greater Bay Area, Shenzhen Polytechnic, Shenzhen, Guangdong, China

**Keywords:** Person re-identification, Context information, Heterogeneous feature fusion, Vision transformer, Convolutional neural networks

## Abstract

Person re-identification plays an important role in the construction of the smart city. A reliable person re-identification system relieves users from the inefficient work of identifying the specific individual from enormous numbers of photos or videos captured by different surveillance devices. The most existing methods either focus on local discriminative features without global contextual information or scatter global features while ignoring the local features, resulting in ineffective attention to irregular pedestrian zones. In this article, a novel Transformer-CNN Coupling Network (TCCNet) is proposed to capture the fluctuant body region features in a heterogeneous feature-aware manner. We employ two bridging modules, the Low-level Feature Coupling Module (LFCM) and the High-level Feature Coupling Module (HFCM), to improve the complementary characteristics of the hybrid network. It is significantly helpful to enhance the capacity to distinguish between foreground and background features, thereby reducing the unfavorable impact of cluttered backgrounds on person re-identification. Furthermore, the duplicate loss for the two branches is employed to incorporate semantic information from distant preferences of the two branches into the resulting person representation. The experiments on two large-scale person re-identification benchmarks demonstrate that the proposed TCCNet achieves competitive results compared with several state-of-the-art approaches. The mean Average Precision (mAP) and Rank-1 identification rate on the MSMT17 dataset achieve 66.9% and 84.5%, respectively.

## Introduction

Intelligent video systems with multi-camera collaborative work play an important role in the real world, which overcomes the view limitations of single and fixed camera monitoring. Among many types of intelligent video systems, person re-identification (ReID) has attracted increased attention in industry and academics (Bai et al., 2020; Luo et al., 2019b; Wang et al., 2020). ReID automatically associates all images of one interest person captured by different camera devices (Zheng, Zheng & Yang, 2017a), which is used to analyze the trajectory of a specific person’s activity in a wide area. It relieves the users from the tedious and time-consuming task of identifying specific pedestrians from enormous numbers of photos and videos, improving efficiency. Additionally, it compensates for the failure of face recognition in the case of long-distance capture. Therefore, ReID has been widely used in various typical applications, including but not limited to, lost person recovery, suspect tracking, and smart business-unmanned supermarkets (He et al., 2021; Luo et al., 2019a; Sun et al., 2018).

Actually, ReID is still an inherently challenging problem (Sun et al., 2018). It mainly relies on the apparent characteristics of the person, which are closely related to appearance such as clothing and posture. However, there are different degrees of differences caused by varying viewing angles, lighting intensity and body pose variations among pictures of the same person, because one person can freely move inside the camera view without any limitation. Specifically, the picture, except for the pedestrian, comprises dynamically shifting and invalid background clutter. Moreover, the complete body even unable to be captured by imperfect pedestrian detection or obstruction. Therefore, how to precisely focus on the body discriminative region while excluding invalid background information is the key to identify all images of the same ID individual (Chen et al., 2020).

To this end, most existing Re-ID methods focus on learning discriminative features. Traditional hand-crafted feature extraction such as the ensemble of local features (Gray & Tao, 2008), fisher vectors (Ma, Su & Jurie, 2012), as wells as metric learning methods such as KISSME (Keep Simple and Direct Metric Learning, Cheyenne, WY, USA) (Liao et al., 2015), XQDA (Cross-Perspective Quadratic Discriminant Analysis) (Koestinger et al., 2012) for constraining the distance relationship between features have finished person re-identification to a certain extent. However, it relies on experience and is not robust to the variable real environment. Advances in computer vision technology provide technical support for ReID. The applications of deep learning algorithms achieve end-to-end recognition and also improve recognition accuracy, which has become a research hotspot. For now, the general attention mechanism based on convolutional neural networks (CNN) is introduced for ReID to guide the network to focus on extracting the essential components (Chen et al., 2019; Chen et al., 2020; Hou et al., 2019), alleviating the issue of pedestrian posture misalignment and clutter. Nevertheless, attention mechanism based on local convolutional operations is not suitable for the capture of global information, preventing the attention module from achieving a more reasonable weight assignment for important pedestrian region features (Zhang et al., 2020). Vision transformer does well in generating global information (Peng et al., 2021), and He et al. (2021) propose to implement the vision transformer to integrate global context for robust person representations. The inherent advantage of vision transformer based entirely on self-attention makes it particularly powerful in capturing long-term dependencies, adaptively giving varying attention to information in different regions from a global perspective, and pushing the model to perceive human body regions (arms, thighs, waist, etc.) in the image. Nevertheless, pure vision transformer lacks inductive bias inherent in convolution without enough ability to extract local structural features, edges and corners of human bodies, resulting in the weak discriminability of features between background and foreground. Thus, it is difficult for pure vision transformer to deal with background clutter and high similarity of attributes between instances. On the contrary, the convolution operation that computes the correlation among neighboring local pixels layer by layer, unlike vision transformer, is naturally suitable for extracting local information. Ascoli et al. (2021) states that localization in convolution operation has certainly reasonable compensations for global dependencies in vision transformer.

Motivated by such observations, in this article, heterogeneous feature based on Transformer-CNN Coupling Network (TCCNet) is proposed to learn the discriminative and robust feature for ReID. TCCNet combines the representational modeling capabilities of CNN and vision transformer to model the global structure and local salient features of human body regions in parallel. TCCNet follows the idea of “divide and conquer” to maintain the advantages of CNN and vision transformer in person re-identification. During local modeling, CNN-branch ignores the spatial relationship between local features (and vice versa). Two bridging modules, *i.e*., the Low-level Feature Coupling Module (LFCM) and the High-level Feature Coupling Module (HFCM), are presented to connect shallow and deep features in the hybrid network. As a result, the vision transformer branch may make full use of the features captured in the CNN branch to activate more attention in the human body region rather than the background, and therefore adaptively discover and align features. The representation extracted by the vision transformer branch can guide the CNN branch in what regions to extract features more effectively.

Extensive experiments are conducted on three commonly used datasets to validate the effectiveness of the method proposed in this article. The results clearly demonstrate that our method outperforms most existing relevant methods. The main contributions of this article are summarized as follows:

(1) A novel heterogeneous network framework, TCCNet, is firstly proposed by using interactive feature propagation for ReID, which is capable of observing highly salient information for pedestrian from a global perspective.
(2) LFCM and HFCM are introduced to enhance the complementarity of the two branches, adaptively aggregate the heterogeneous features, and significantly reduce the influence of background noise on the model.
(3) The proposed TCCNet achieved competitive results on the publicly available datasets, *i.e*., Market1501 (Zheng et al., 2015), MSMT17 (Wei et al., 2018) confirming its ability to effectively learn discriminative features in complicated situations.

## Related works

ReID models are mainly based on deep learning, as represented by CNN and vision transformer. In this section, we overview the relevant methods, which are of great enlightenment for our work.

### CNN-based person re-identification

Zheng, Zheng & Yang (2017a) proposed a CNN-based Identity Embedding (IDE) model to construct Re-ID as a multi-classification problem by treating each pedestrian ID as a different class during the training process. After that, most of the Re-ID methods (Luo et al., 2019a; Sun et al., 2018; Zhong et al., 2017) based on representation learning are developed from this IDE model. Since CNNs were initially applied to basic tasks such as image classification, early integration of advanced CNNs into ReID task is usually based on the direct learning of image global features, which is obtained from the whole image as the first choice (learning the best global features from the whole task features). However, such a network model tends to focus more on the part that contributes most to the classification performance, instead of considering all features of the person (Chen et al., 2020). Importantly, the neglected parts often have recognition value as well. So, the global features obtained in the above way are sensitive to the redundant background, and invalidly cope with the pedestrian pose misalignment problem.

To alleviate the influence of background clutter on global features, most existing pedestrian re-identification methods can be classified into three categories. (1) One way is to transform deep-level features into explicit body parts representation with the help of location information extracted by the pose estimation model or the human key point estimation network (Gao et al., 2020; Sarfraz et al., 2018; Su et al., 2017). Unfortunately, state-of-the-art pose estimation networks are not perfect. Such methods are easily limited by these additional auxiliary models. (2) Another type of methos is mainly based on attentional mechanisms, and typical schemes including ABD-Net (Chen et al., 2019), ConsAtt (Zhou et al., 2019), and SONA (Xia et al., 2019), which capture the relationship between different convolution channels, multiple feature maps, layers, different body parts/regions, and even multiple images to enhance the model’s learning of features in pedestrian regions. However, the attention in these works is embedded deep into the CNN network. Due to the inherent limitations of the convolution operator perceptual field, the attention obtained by local convolution in this way ignores the global information and implicit relationships of the images, thus, limiting the ability of attention to learn correlations between features. (3) Multi-branch networks are originally designed to allow the network to learn various features from different perspectives. For existing network models employing multiple branches (Ning et al., 2020; Wang et al., 2018; Zhang et al., 2019), the features of each branch are either replicated from the higher levels of the global branch or extracted from a homogeneous network, leaving the model lacking structurally diverse differential features.

The feature extraction of the proposed network for key regions is learned directly from the data and context and does not rely on manually defined partial, partial region proposals, nor on pose estimation models.

### Transformer-based person re-identification

Transformer has become increasingly popular for computer vision tasks due to its superior interaction capabilities with global self-attention. He et al. (2021) designed a pure transformer architecture combining additional camera encoded information and jigsaw patches module to learn discriminative features. Zhang et al. (2021) took advantage of transformer ability to capture global context to aggregate multiscale features between different layers of CNN and align part features by self-attentiveness. The potential of pure transformer network structure for image based ReID is demonstrated, however, transformer is susceptible to the interference of patch neighborhood detail loss and cannot capture local fine-grained information (Yuan et al., 2021). It may limit the ability to discriminate pedestrians with similar attributes. In addition, the transformer does not have the hierarchical structure of CNN, which makes it difficult to generate local spatial contexts at each level. Moreover, the transformer lacks the inductive bias (Dai et al., 2021; Dosovitskiy et al., 2021) inherent to the CNN branch and has a strong dependence on the amount of data. One of the pain points in pedestrian re-identification research is that there is not enough data to satisfy this exorbitant dependence.

Both the vision transformer and CNN have complementary advantages and limitations in dealing with visual representations. Many current works try to combine CNN and vision transformer in different ways (Li et al., 2021; Peng et al., 2021; Zhang et al., 2021). Inspired by the above work, we design heterotypical networks and combine the complementarity of varying features extracted from different paradigms in ReID to help enhance model performance.

## Methodology

### Overview

**Figure 1:**
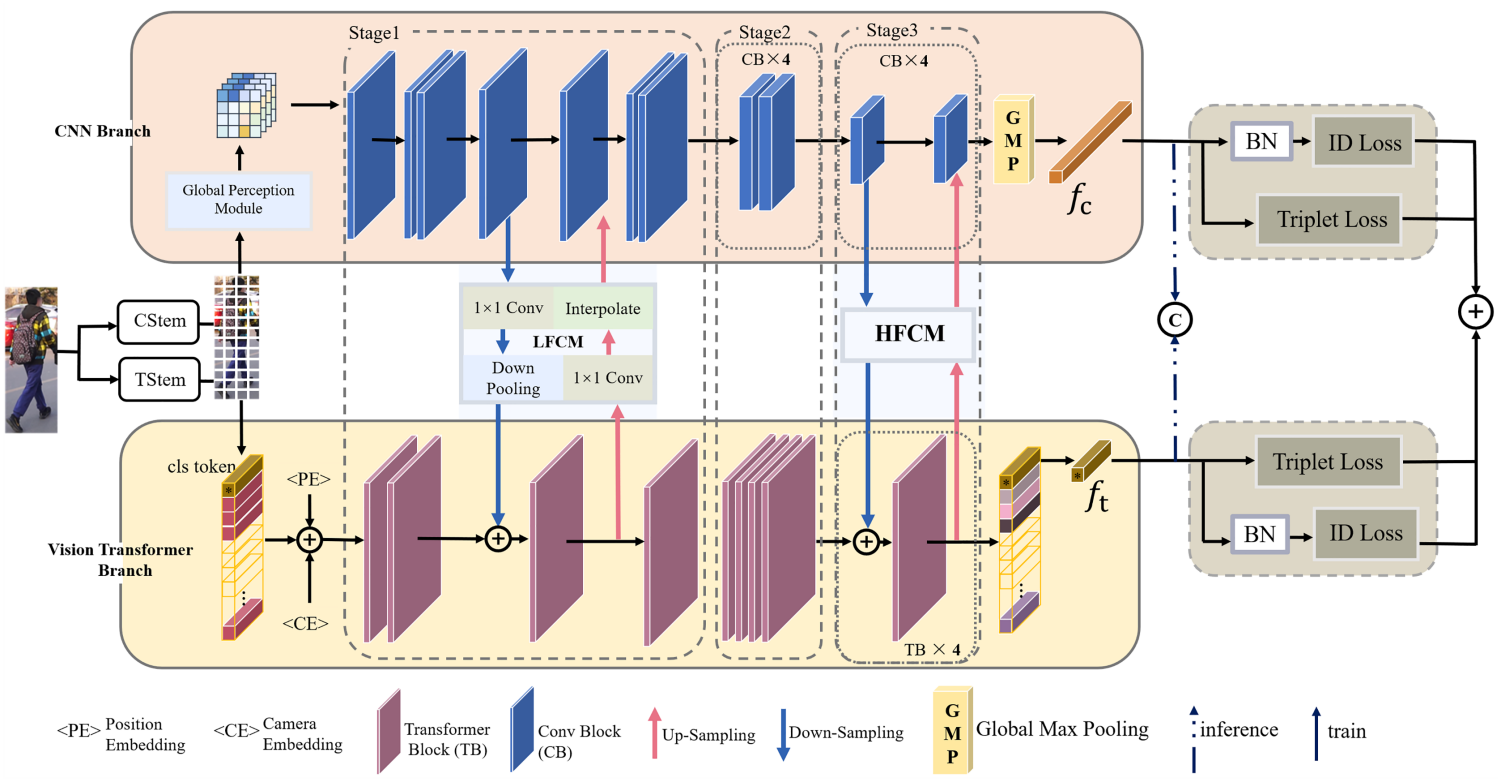
Illustration of the proposed Transformer-CNN Coupling Network (TCCNet).

The proposed framework (TCCNet) is shown in Fig. 1, which consists of three main parts: heterogeneous feature extractor based on CNN-transformer, low-level feature coupling module (LFCM), and high-level feature coupling module (HFCM).

In terms of the overall architecture, TCCNet is a two-branch parallel network taking the advantages of both vision transformer and CNN. The vision transformer branch captures substantial long-range dependencies by stacking multi-headed attention modules, primarily to learn the potential spatial topological of pedestrians in images. The CNN branch aggregates local significant semantic information in a hierarchical manner through deep stacking of convolutional operations. Maximizing the structural advantages of complementary CNN and vision transformer reduces the influence of background clutter toward the representation learning, and obtains more discriminative representations on pedestrian body regions, alleviating the problem of feature misalignment. TCCNet proposed in this article is not an iterative stacking of two branches, but a symmetric interaction network using two bridging modules, *i.e*., LFCM and HFCM, to achieve association (*i.e*., features that flow into the transformer-branch also feed into the transformer-branch, vice versa). LFCM can enhance the ability of CNN branches to perceive global information at a shallow level, while alleviating the lack of discriminative properties of transformer-branch for human boundaries due to the lack of fine local details, and enabling the complementarity of overall pedestrian features and local fine-grained features such as texture and color. The HFCM is used to aggregate the semantic information of different preferences extracted from transformer-branch and CNN branches in depth, so that the final pedestrian representation is structurally diverse and semantically rich. Finally, the dual-stream network is trained end-to-end with duplicate losses jointly. In the testing phase, the final outputs of the two branches are connected for identification, and the dual-stream network is trained end-to-end with duplicate losses jointly. During the testing period, the final outputs of the two branches are concatenated for identification.

### Heterogeneous feature representation based on CNN-Transformer

**CNN-based salient feature extractor.** The CNN-branch is structured with a feature pyramid. The whole branch is divided into three stages, denoted as ${\rm{Stag}}{{\rm{e}}_1}$, ${\rm{Stag}}{{\rm{e}}_2}$ and ${\rm{Stag}}{{\rm{e}}_3}$, as shown in Fig. 1. If the input feature of one stage is defined as $x \in {\mathbb R}^{H \times W \times C}$ (where *C*, *H* and *W* denote the number of the channel, width and height of features, respectively), the output feature of this stage is $x \in {\mathbb R}^{{H \over 2} \times {W \over 2} \times (2 \times C)}$. Corresponding to the three stages, three hierarchical feature maps with different resolutions can be obtained.

Each stage is composed of a different number of convolution blocks, set to 7, 8, and 8, experimentally. Except for the first convolution block in ${\rm{Stag}}{{\rm{e}}_1}$, all subsequent convolution blocks appear in pairs. The outputs of the convolution blocks at the same stage have the same feature resolution. The convolution block is a stack of three convolutional layers (1 × 1, 3 × 3, 1 × 1). The CNN-branch ends with a batch normalization layer and a global max pooling layer to generate one vector as pedestrian locally salient feature.

**Transformer-based interrelated feature extractor.** For the vision transformer branch, the input image passed the Transformer Stem (TStem) unit is first represented as a sequence of N patches $\left\{ {{x_i}|i = 1,2, \ldots ,N} \right\}$ and modify them as the input of transformer blocks. Each patch is defined as ${x_i} \in {\mathbb R}^{P \times P \times C}$, where P and C respectively denote the spatial dimension and the number of channels. The total number of patches N depends on kernel size, stride, and size of the image. Here, N can be easily calculated using the Eq. (1). $H,W$ are height and width of an image, respectively, equal to P. ${K_H}$, ${K_W}$ are height and width of the kernel, and S is kernel stride.

(1) $$N = \left\lfloor {{{H - {K_H}} \over S} + 1} \right\rfloor \times \left\lfloor {{{W - {K_W}} \over S} + 1} \right\rfloor.$$

The patches are then projected into N E-dimensional vectors using the learnable embedding ${\cal F}$. After that, a learnable class token ( ${x_{cls}}$), which can be used as a discriminative representation, is concatenated with the above N E-dimensional embedding representation ( ${\cal F}\left( {{x_i}} \right)$). Pedestrian images with the same ID are taken across cameras, resulting in a data distribution gap in the embedding subspace. In addition to introducing a learnable position embedding (PE) for each patch to learn the spatial information between blocks and a learnable camera embedding (CE) He et al. (2021) added to learn the camera information, which aims to reduce the bias caused by changing camera settings. The sequence of embedded patches ${z_0}$ is:

(2) $${z_0} = \left[ {{x_{cls}};{\cal F}\left( {{x_1}} \right);{\cal F}\left( {{x_2}} \right); \ldots ;{\cal F}\left( {{x_N}} \right)} \right] + PE + CE,$$

where ${x_{cls}} \in {\mathbb R}^{N \times E},{\cal F}\left( {{x_1}} \right) \in {\mathbb R}^{N \times E},PE \in {\mathbb R}^{\left( {N + 1} \right) \times E},CE \in {\mathbb R}^{\left( {N + 1} \right) \times E}.$

Then, ${z_0}$ is feed to *L* repeated transformer blocks. Every transformer block consists of layer normalization (LN), multi-head self-attention (MSA), and multilayer perceptron (MLP) in sequential order. The input and output of the multi-headed attention layer are connected with residuals and layer normalization.

(3) $$z_l^\prime = MSA\left( {LN\left( {{z_{l - 1}}} \right)} \right) + {z_{l - 1}}\;\;\;l = 1, \ldots ,L,$$

(4) $${z_l} = MLP\left( {LN\left( {z_l^\prime } \right)} \right) + z_l^\prime\;\;\;\;\;\; l = 1, \ldots ,L.$$

The feature *z* is first passed to the MSA layer in every transformer block. MSA can be expressed as Eq. (5).

(5) $$MSA\left( z \right) = Concat\left( {{{{H}}_1},{{{H}}_2}, \ldots ,{{{H}}_h}} \right){{{W}}^o},$$

where h is the number of heads in MSA, and concat (∙) denotes stacking on the embedding representation dimension of the patch embedding. Each header ${{{\rm H}}_i}(i = 1,2, \ldots ,h)$ can be expressed as follows:

(6) $$Attention\left( \cdot \right) = softmax\left( {{{Q{K^T}} \over {\sqrt d }}} \right)V,$$

$\matrix{ {Q = z{{W}}_i^Q} \cr {K = z{{W}}_i^K} \cr {V = z{{W}}_i^V} \cr }$

where *Q, K, V* are generated by three completely different linear projections, respectively. It is seen that *Q, K, V* are expressed as ${{W}}_i^Q,{{W}}_i^K \in {\mathbb R}^{E \times {d_k}}$, ${{W}}_i^V \in {\mathbb R}^{E \times {d_v}}$, which means they are more expressive. Attention (∙) is a function used to calculate the relevance and importance of the patch embeddings. $\sqrt d$, the scaling factor, is used to ensure numerical stability through normalization.

The MLP contains a two-level linear projection and a GELU activation function. It is denoted as:

(7) $$MLP\left( z \right) = {{{W}}_2}\sigma \left( {{{{W}}_1}z} \right),$$

where ${{{W}}_1}$, ${{{W}}_2}$ are the parameters of the two linear projections and σ is the GELU activation function.

The last class token of representation captured by the vision transformer-branch serves as a representation of the overall pedestrian structure.

**Separated double stem unit.** Making full use of the feature base captured by lower of the network will achieve greater discriminability. We advocate the idea of using the Separated Double Stem, the transformer Stem (TStem) and CNN Stem (CStem), to perform down-sampling on the input image. In other words, TStem and CStem are constructed by two different convolution operations. CStem and TStem are both 16 × 16 filter with stride of 12 to get overlapping patches. The difference is that CStem contains 64 filters, while TStem contains 768 filters. Overlapping patches can introduce correlations between neighboring blocks, which in turn reduces the impact of the structure being corrupted and learns more local detail information. Heterogeneous networks have different ways of processing representations (Woo et al., 2018), leading to varying requirements for their feature response maps in the lower layers of the network. Specifically, the vision transformer-branch captures the holistic information from a global perspective at the low-level of the network. In contrast, the CNN-branch gradually learns global features as the depth of the network increases. Avoiding the limitations and maximizing the advantages of both network architectures is essential. The experimental results and analysis about this part are shown in “Analysis of separated double stem unit”.

### Heterogeneous feature coupling

**Global perception module.** This is a plain module composed of only a fully connected layer, but the contribution of it is not negligible. Essentially, it is to allow the CNN branch to collect perceive information from a larger region ahead of the LFCM. Invalid features in complex backgrounds can have a suppressive effect on extracting discriminative pedestrian information. Features captured by convolution with limited receptive fields may compromise the representation obtained by transformers from self-attention, which leads to a limited ability to interact with heterogeneous features. Global Perception Module makes a contribution to alleviate this problem.

**Figure 2:**
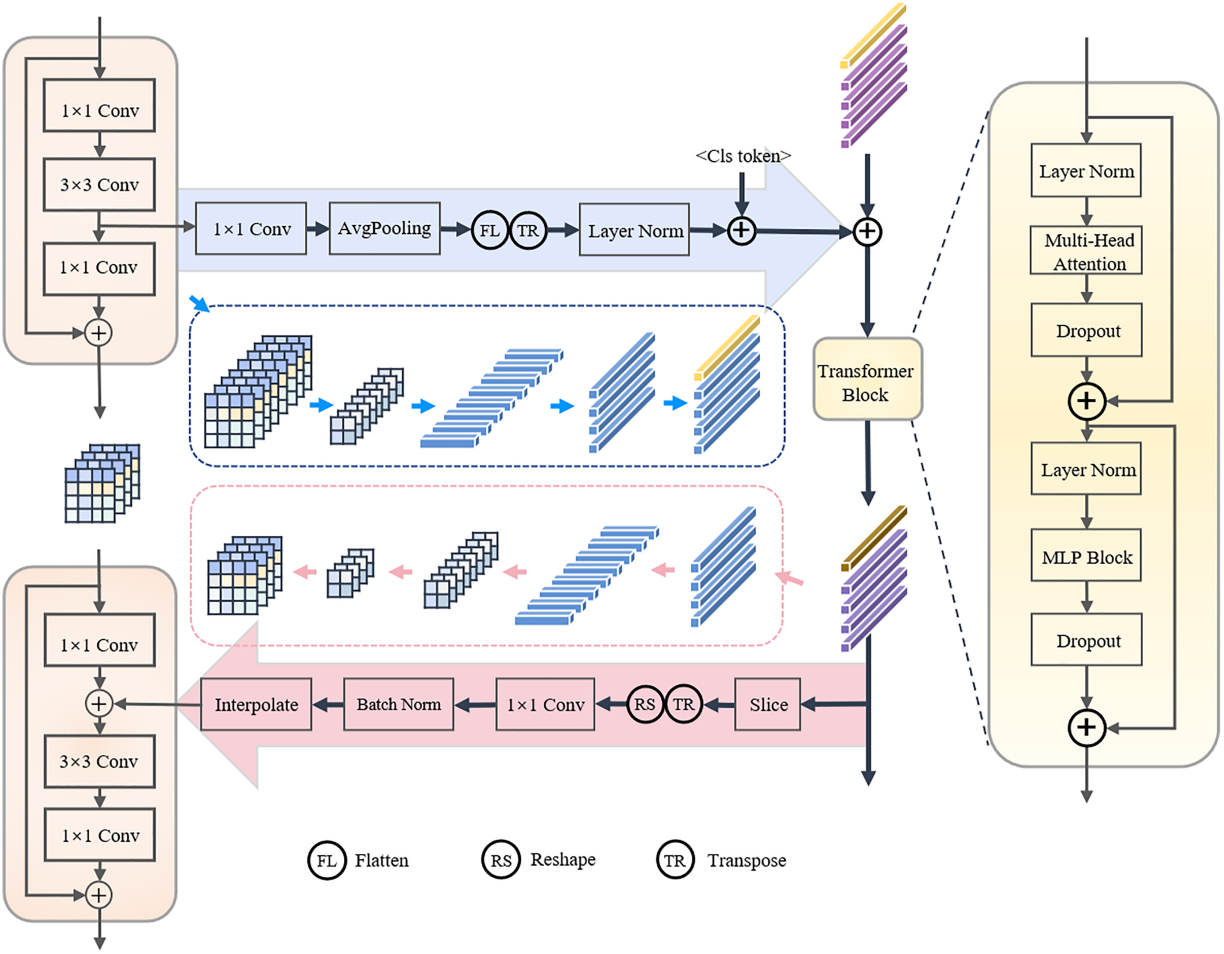
Feature coupling module.

**Feature coupling module.** There are significant differences between CNN and transformer branches in the way of process representations and the ability to integrate semantic information in high-level. The CNN-branch strictly obeys the process of converging global features from local features. In contrast, both local and global information are captured in the lower and higher layers of transformer. As a result, the difference between the representations in the two branches increases layer by layer. In order to obtain a semantically rich pedestrian representation, the complementarity of the two branches needs to be enhanced. To ensure the effectiveness of the heterogeneous feature fusion, the strategy of integrating all layer features in a very extreme way is discarded. The heterogeneous feature coupling module, which is shown in Fig. 2, is applied to the low and high layers of the TCCNet (where correspond to the stage1 and stage 3), respectively. Specifically, inspired by conformer, the features extracted by the CNN-branch are dense three-dimensional, and the embedding representation dimension of the transformer-branch is two-dimensional. When CNN-branch features flow into transformer-branch, the channel dimension should first be transformed with a 1 × 1 convolution. The features in different channels can be understood as the response of the input image towards different feature patterns. There is a semantic gap between the features extracted from the vision transformer and CNN branch, so the features of the CNN branch use the global average pooling operation to retain some features that best express the image, and these focal features are normalized by Layer Normalization and then converged to the vision transformer branch to achieve detailed features in the key region. The representation obtained from the Transformer is also fed back to the CNN-branch by first aligning the channels with a 1 × 1 convolution, and then adding them to the feature of the CNN-branch after Batch Normalization and interpolation to align the spatial resolution of the features with the CNN-branch.

### Supervision learning with duplicate loss

**Loss function.** In this article, the network is supervised using cross-entropy loss with BNNeck (Luo et al., 2019a) (called ID loss in this article) and triplet loss. ID loss is used for representation learning that can motivate the network to learn the implicit relationships that exist among identities. The purpose of the person re-identification task is to minimize the distance among the same identities while maximizing the distance among different identities. The core principle of triplet loss is to pull the distance between positive sample pairs and push away the distance between negative sample pairs. Therefore, triplet loss is introduced to explicitly learn the required similarity measure for recognition. ID loss and triplet loss have complementary advantages in learning discriminative pedestrian representations (Zheng, Zheng & Yang, 2017a).

The ID loss is defined as:

(8) $${L_{ID}} = - {1 \over N}\sum\nolimits_{i = 1}^N {{q_i}} \log \left( {{p_i}} \right),$$

where ${q_i}$ denotes the ground-truth label, ${p_i}$ is the probability that the network predicts identity i, and *N* is the number of images in each batch of the input.

The triplet loss is defined as

(9) $${L_{tri}} = \log \left[ {1 + \exp \left( {{d_{pos}} - {d_{neg}}} \right)} \right],$$

where ${d_{pos}}$ and ${d_{neg}}$ denote the distances of positive and negative sample pairs, respectively.

The loss of a single branch can be expressed as

(10) $${L_{CNN}} = {L_{transformer}} = \alpha {L_{tri}} + \left( {2 - \alpha } \right){L_{ID}},$$

where *α* is used to balance the two losses.

**Duplicate loss.** To motivate the two branches to learn their preferred features, we optimize the network by constructing duplicate loss, which is a separate classifier for each branch, as shown in Fig. 1. The total loss is expressed as

(11) $${L_{total}} = \lambda {L_{transformer}} + \left( {1 - \lambda } \right){L_{CNN}},$$

where λ is used to balance the losses of the two branches. In this article, *α* is set to 1 and λ is set to 0.5 based on experiments to optimize the network model by minimum ${L_{total}}$.

### Similarity measurement

During the similarity measurement, the features of the images in the Query and Gallery sets are first extracted with the trained model, and then the recognition distance matrix is obtained by calculating the Euclidean distance between the two feature sets in the embedding space. The image with the smallest distance is considered as the most likely candidate person.

Given a set of test images is divided into Query and Gallery, with Query denoted as $Q = \left\{ {{q_i}} \right\}_{i = 1}^m$ and Gallery denoted as $G = \left\{ {{g_i}} \right\}_{i = 1}^n$, the feature vectors corresponding to *Q* and *G* are denoted by ${F_Q}$ and ${F_G}$, respectively, and shown as follows:

(12) $${F_Q} = {\rm{ }}\left[ {{f_{q1}},{f_{q2}}, \cdots ,{f_{qm}}} \right]{\rm{ }} \in {^{m \times d}},$$

(13) $${F_G} = \left[ {{f_{g1,}}{f_{g2}}, \cdots ,{f_{gn}}} \right] \in {\mathbb R}^{n \times d},$$

where $m$ denotes the number of images contained in Query, $n$ denotes the number of images contained in the Gallery, and $d$ denotes the image feature dimension. Because the Euclidean distance is the simplest and easiest to understand, the distance between Query image ${q_i}$ and Gallery image ${g_i}$ is expressed as:

(14) $$d\left( {{q_i},{g_i}} \right) = {\left\| {\overrightarrow {{f_{qi}}} - \overrightarrow {{f_{gi}}} } \right\|_2} = \sqrt {{{\left( {\overrightarrow {{f_{qi}}} - \overrightarrow {{f_{gi}}} } \right)}^T}\left( {\overrightarrow {{f_{qi}}} - \overrightarrow {{f_{gi}}} } \right)} ,\quad i \le m,j \le n,$$

where ${f_{qi}}$ and ${f_{gi}}$ denote the features obtained by inputting images ${q_i}$ and ${g_i}$ into the TCCNet network.

## Experiment and result analysis

### Datasets

The proposed model is evaluated on two public datasets, *i.e*., Market1501 (Zheng et al., 2015) and MSMT17 (Wei et al., 2018) datasets. Market1501 is a dataset consisting of 32,668 images of 1,501 pedestrians captured by six different cameras. MSMT17 is captured by 12 outdoor cameras and three indoor cameras, totally 126,441 images of 4,101 pedestrians. It is a large dataset closer to the real scene and is more reflective of the model performance. The details of two datasets are shown in Table 1.

**Table 1:** Setting of person re-identification datasets.

Dataset	Market-1501	MSMT17
Identities	1,501	4,101
Identities (training)	751	1,041
Identities (testing)	750	3,060
Bounding box (training)	12,936	23,621
Bounding box (query)	3,368	11,659
Bounding box (gallery)	12,936	82,162

### Implementation details and evaluation metrics

All experiments were implemented on NIVDIA A100-SXM4-40G GPUs. Initial weights of Transformer were pre-trained on ImageNet-21K and then fine-tuned on ImageNet-1K.

Before training, the resolution size of the input image was adjusted to 256 × 128, and the training batch was set to 64, including 16 different pedestrians and four images each. The data were augmented by random horizontal flipping, padding, random cropping, random erasing and normalization strategies. The total number of training epoch was set to 150. The model was optimized using SGD, where the weight decay factor was set to 1e–4 and the momentum was set to 0.9. The initial learning rate was 0.009 with cosine learning rate decay.

In this article, the experimental performance was evaluated using the cumulative matching curve (CMC) at Rank-1 and the mean average precision (mAP). Compared to CMC. The mAP is a more comprehensive evaluation metric, which is calculated as:

(15) $$mAP = {1 \over m}\sum\nolimits_{i = 1}^m A {P_i},$$

where $m$ is the number of query images in the query set; $A{P_i}$ is the average precision of the i-th query image.

### Analysis of separated double stem unit

**Separated double stem unit.** In order to verify the effectiveness of the proposed Separated Double Stem Unit, two sets of experiments are carried out. The experimental results are shown in Table 2. Shared Single Stem means that the CNN-branch and vision transformer branch share weights, while Separated Double Stem means that the CNN branch and vision transformer branch each have independent stem modules. The experimental results show that the use of large convolutional kernels for overlapping to slice the patches achieves the best results on both sets of experiments, and separated double stem achieves the best experimental results among all the experimental results by this slice.

**Table 2:** Comparison of different designs of stem modules.

Method	Shared single stem		MSMT17	
			mAP (%)	Rank-1 (%)
TCCNet (shared weight)	7 × 7, stride 2		57.3	78.4
	16 × 16, stride 16 (non-overlapping)		58.9	77.8
	16 × 16, stride 12 (overlapping)		62.8	81.2
	**Separated double stem**			
	**TStem**	**CStem**		
TCCNet (w/o shared weight)	16 × 16, stride 16	4 × 4, stride 4	61.6	80.4
		8 × 8, stride 8	61.6	79.6
		16 × 16, stride 16	62.6	83.5
	16 × 16, stride 12	4 × 4, stride 3	63.1	81.1
		10 × 10, stride 6	62.9	81.1
		16 × 16, stride 12	66.9	84.5

Experiments in the Separated double stem section show that TStem and CStem achieve optimal results in over-lapping and non-overlapping modes using convolutional kernels of the same size. Convolutional kernels with the same size ensure the consistency of the perceptual fields (Sun et al., 2018; Zheng, Zheng & Yang, 2017b) of CStem and TStem, allowing the two branches to align features efficiently and facilitating feature fusion in the LFCM.

**Analysis of global perception module.** To enable the CNN branch to collect information over a relatively large area, the feature space resolution is adjusted using an up-sampling operation. In Table 3, different up-sampling strategies are compared. It is found that the linear approach provides a more significant contribution to the improvement of the model performance.

**Table 3:** Experiments of global perception module.

Method	MSMT17	
	mAP (%)	Rank-1 (%)
Convolution	59.1	77.2
Transpose conv	64.8	82.4
Interpolation	63.8	81.3
Pixel shuffle	65.7	83.2
Linear	66.9	84.5

### Analysis of feature coupling module

**Analysis of LFCM.** The first stage of the CNN branch contains three pairs of convolutional blocks, which means that, in this phase alone, there are multiple fusion modes. In order to design an effective low-level feature coupling module, we compare all possible effects of different feature coupling in the first stage, which is shown in Table 4. Here, we define whether the three pairs of convolutional blocks in the first stage interact with the transformer-branch with features as { ${{\rm{b}}_1},{{\rm{b}}_2},{{\rm{b}}_3}$}, and ${{\rm{b}}_{\rm{i}}} = 1$ indicates that the i-th pair of the convolutional block in the CNN branch interacts with representation in the transformer-branch.

**Table 4:** Comparison of different layer schemes of LFCM.

Index	Layer	MSMT17	
		mAP (%)	Rank-1 (%)
1	{000}	64.5	82.4
2	{001}	64.5	82.0
3	{100}	64.2	82.2
4	{101}	65.0	82.6
5	{010}	66.9	84.5
6	{011}	65.4	83.3
7	{110}	66.0	83.3
8	{111}	62.8	81. 8

From the experimental results in Table 4, it is seen that the fusion method of index 5 is optimal. Moreover, the performance of the second pair of convolutional block features for interaction (index 5, 6, 7) is superior to that without interaction (index 1, 2, 3, 4), except for index 8, which shows the necessity of feature interaction between the second pair of convolutional blocks in the CNN-branch and the transformer-branch.

The reason why the performance of all three pairs of residual blocks with interaction (index 8) is worse than the performance of all other choices may be that it produces redundant features, overemphasizes certain non-accurate regions, misleads the learning of the network, and the model performance decreases.

**Figure 3:**
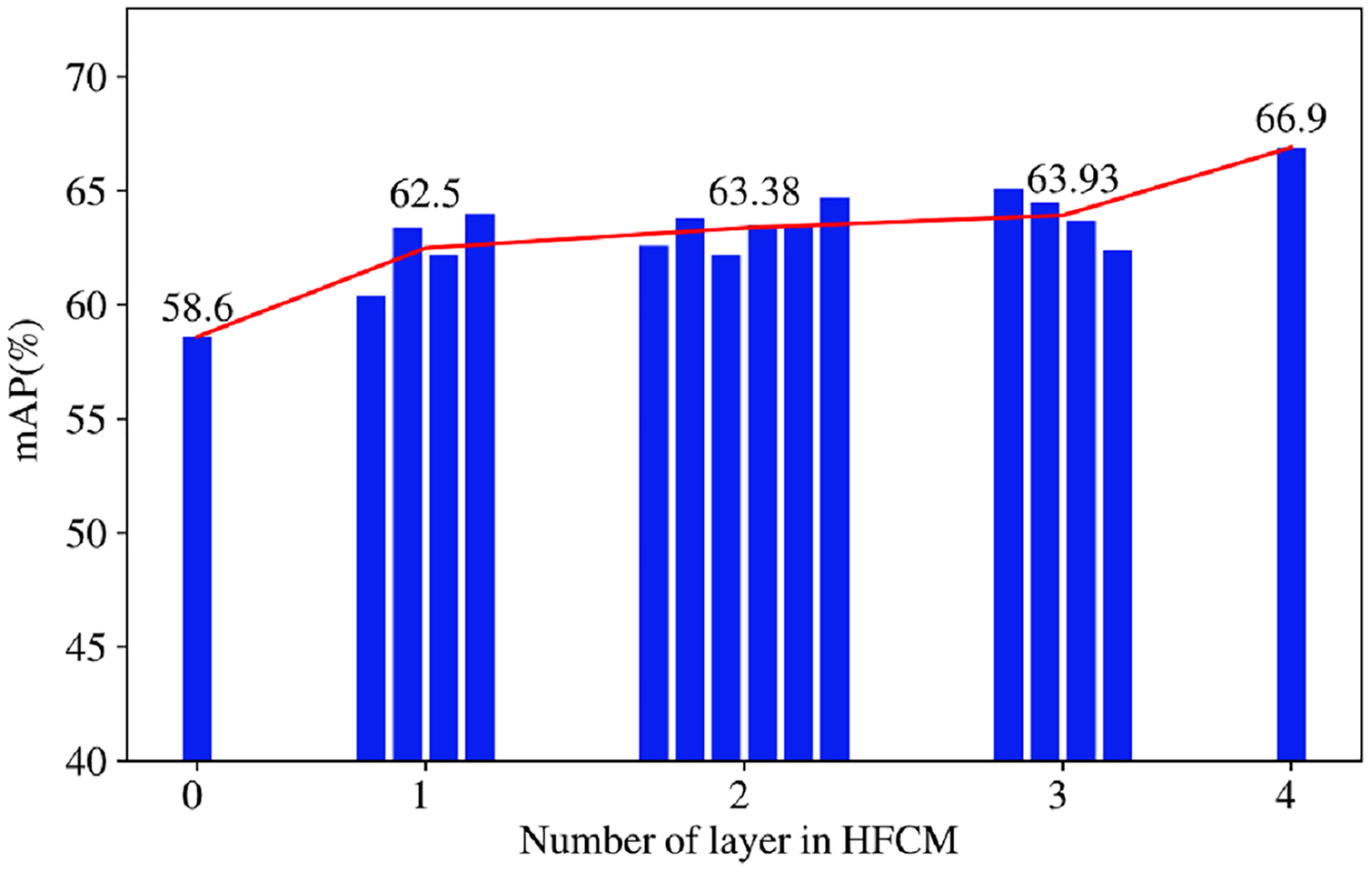
Experiments of the layer number of HFCM.

**Analysis of HFCM.** Quantitative experiments are performed to find the appropriate number of feature coupling in high-level layers. The third stage of the network has four convolutional layers, which can be classified into four categories according to the number of fusion layers and subdivided into 16 options. The detailed results corresponding to this are shown in Table 5. The histogram in Fig. 3 represents the model performance with the different number of fusion layers. The line graphs indicate the mean value of mAP corresponding to each class of fusion layers. As shown in Fig. 3, it is found that model without coupling of high-level features are not robust enough, and performance of the model is the worst. The model performance rises gradually with increase of the number of coupling layers in HFCM. The best performance can be achieved when the number of fusion layers is set to four (*i.e*., the features of third stage are all fused). The HFCM can comprehensively use the overall features and local discriminative features of pedestrians to further locate the high response features of pedestrians on the basis of getting the complete features of pedestrians, which increases the structural diversity required for the model to learn the differential features. As a result, the final pedestrian representation has more valid information and can improve the generalization ability of features.

**Table 5:** Comparison of different layer schemes of HFCM.

Number	Layer	MSMT17	
		mAP (%)	Rank-1 (%)
0	{0000}	58.6	78.2
1	{0001}	60.4	79.2
	{0010}	63.4	80.8
	{0100}	62.2	80.2
	{1000}	64.0	81.4
2	{0011}	62.6	80.5
	{0110}	62.2	80.3
	{1100}	64.7	81.9
	{1001}	63.5	81.3
	{1010}	63.5	81.1
3	{0111}	65.1	82.7
	{1110}	63.7	81.4
	{1101}	64.5	82.3
	{1011}	62.4	80.4
4	{1111}	66.9	84.5

**Ablation experiments of heterogeneous feature coupling modules.** According to the experimental results in Table 6, with the same data augmentation and tricks (Luo et al., 2019a), a large gap between the model capacity of the ViT series and the CNN series can be observed, but the same gap in performance is obvious. The mAP achieves 56.7% using ResNet50 as the baseline network (ResNet50-BOT) and 61.8% using vision transformer as the baseline network (ViT-BOT). The mAP of our proposed TCCNet can reach 66.9%, which indicates that the heterogeneous network structure extracts comprehensive and highly significant features, largely reducing the effect of background noise on the model, thus achieving improved model performance. Furthermore, we set the deeper ResNet101 network as the backbone network. It brings some performance improvement (+2.1%/+0.8%) compared with ResNet50-BOT, but it is still far behind our TCCNet, indicating that the extra branch parameters are not the key contributors to the improvement, but rather the well-designed network architecture. We compare the inference times of several other models with ResNet50-BOT. Although it is larger than other models in capacity, the TCCNet model is superior to several other models in terms of performance, and we will further reduce the model params.

**Table 6:** The ablation study of TCCNet. Inference time is represented by comparing each model to ResNet50-BOT as only relative comparison.

Method	Params (M)	Inference time	Module		MSMT17	
			LFCM	HFCM	mAP (%)	Rank-1 (%)
ResNet50-BoT	23.3	1×	×	×	56.7	79.2
ResNet101-BoT	42.5	1.47×	×	×	57.6	80.1
ViT-BoT/s = 16	92.7	2.11×	×	×	61.0	81.8
ViT-BoT/s = 12	92.7	3.34×	×	×	64.4	83.5
TCCNet (*Ours*)	101.2	5.53×	√	×	58.6	78.2
			×	√	65.2	83.1
			√	√	66.9	84.5

From the experimental results in Table 6, it is very essential for our model that HFCM and LFCM, and removing either module will cause a degradation in model performance. The LFCM and HFCM greatly enhance the global perception of local features of the pedestrian and the local details of the global representation of the network.

### Analysis of duplicate loss

**Figure 4:**
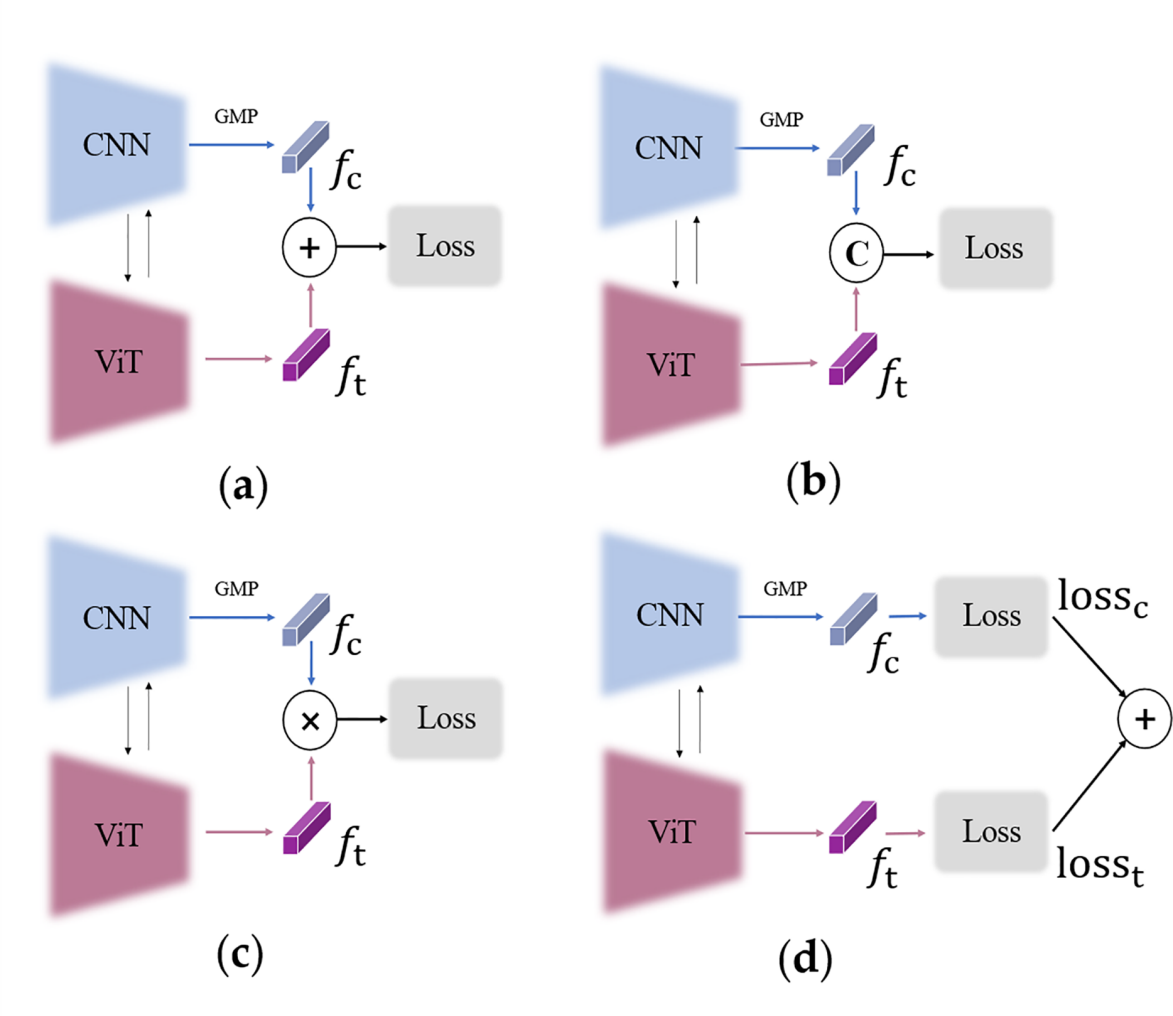
Supervision strategy.

**Choice of supervision strategy.** Supervision strategy directly affects the performance of the heterogeneous networks with joint training. Several different supervision strategies and their corresponding feature representations at test stage are examined. Figs. 4A–4C represent **s**ingle-loss learning. That is, the losses shown in Eqs. (8) and (9) are calculated once for the two-branch fusion feature, and then the two obtained loss values are summed, and the gradient is back-propagated to optimize the network. There are three ways of feature fusion in the single-loss strategy: including element-by-element summation (a), concatenate (b), and element-by-element multiplication (c). Fig. 4D illustrates the duplicate-loss joint learning process. The losses shown in Eq. (10) are computed once for each branch, and the four obtained loss values are summed to optimize the two branches. From the experimental results in Table 7, we can observe that the duplicate-loss joint training strategy performs better and the single-loss training approach is not effective in further improving the model. This is because the duplicate-loss can drive both branches to highlight their own strengths and learn their preferred features. In contrast, it is difficult to train the network stably with only single-loss constrained heterogeneous features.

**Table 7:** Performance for different supervision strategies shown in Fig. 5.

Index	Train	Test	MSMT17	
	Strategy	Final feature	mAP (%)	Rank-1 (%)
(a)	Single-loss	Add	64.1	82.4
(b)	Single-loss	Concat	64.6	83.0
(c)	Single-loss	Multiply	56.6	75.4
(d1)	Duplicate-loss	Add	64.2	81.8
(d2)	Duplicate-loss	Concat	66.9	84.5

**Figure 5:**
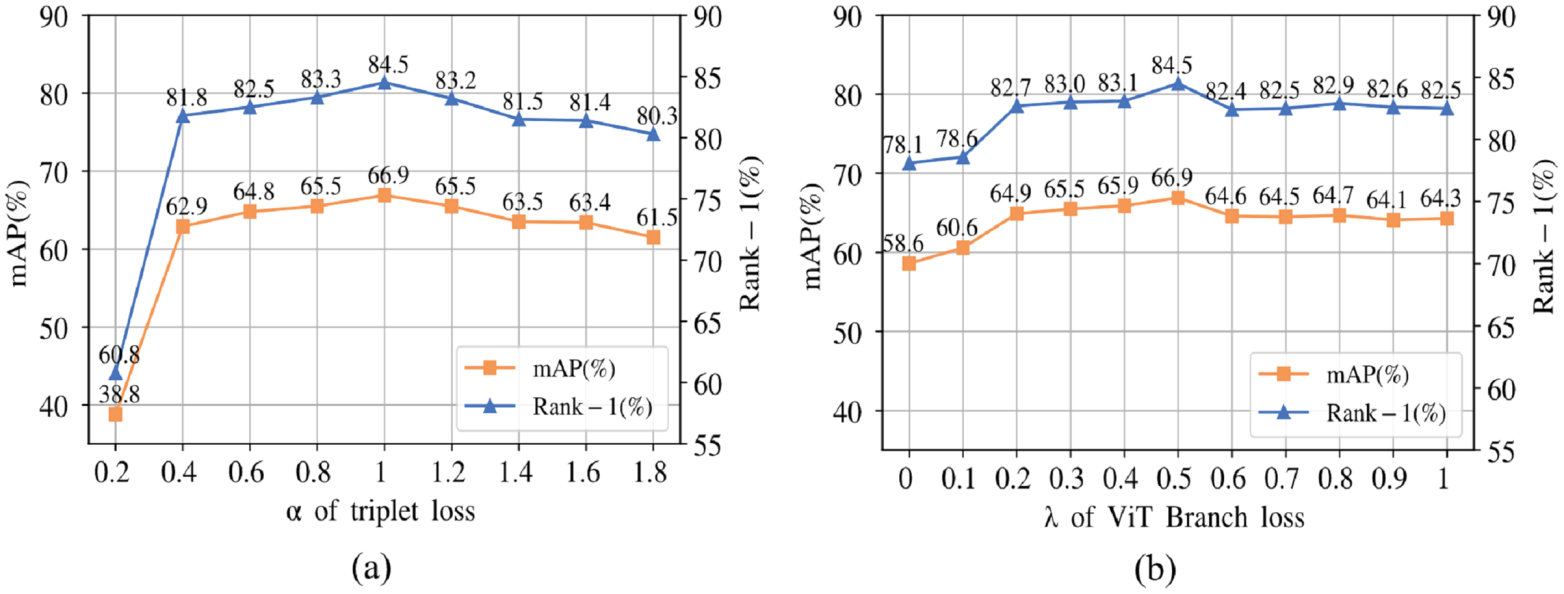
Graphical representation of parameter sensitivity in the loss function.

**Experiment with parameter sensitivity.** The α defined in Eq. (10) is used to represent the weight of the triplet loss in each branch, and λ defined in Eq. (11) is used to represent the weight of the vision transformer branch loss. λ is selected from 0.1 to 1, taking a value every 0.1 interval. We fix each λ and vary α from 0.2 to 1.8, taking a value every 0.2 intervals. Table 8 shows the best performance produced by taking fixed values of λ, corresponding to one α. The total loss where λ is equal to 0.5 and α is equal to 1 has a better performance.

**Table 8:** Experiments of parameter sensitivity of the loss function.

$\left( {{\bf{\lambda }},{\bf{\alpha }}} \right)$	MSMT17	
	mAP (%)	Rank-1 (%)
$\left( {0.1,1.2} \right)$	64.9	83.2
$\left( {0.2,1.4} \right)$	65.1	83.3
$\left( {0.3,1.6} \right)$	65.5	83.5
$\left( {0.4,1.4} \right)$	65.5	83.1
$\left( {0.5,1.0} \right)$	66.9	84.5
$\left( {0.6,1.0} \right)$	65.9	83.1
$\left( {0.7,1.2} \right)$	65.7	83.1
$\left( {0.8,1.2} \right)$	65.5	82.9
$\left( {0.9,0.6} \right)$	64.6	81.7

Figure 5 provides the graphical representation. (a) is the performance of the model with varied α when λ is fixed at 0.5. When α is set to 0.2, the performance of the model produces dramatic decreases. Since triplet loss explicitly learns the similarity metric needed for recognition. As the value of grows, the performance improves. The model performance decreases when α is more than 0.5. ID loss considers the variability between one sample and all samples, making the features near the classification layer more focused on the differences in training identities. The model can be driven to explore useful detailed information in the limited information available, and ID loss is indispensable in person re-identification tasks. (b) in Fig. 5 indicates the trend of λ when α is equal to 1. As the λ weight increases, the accuracy of pedestrian recognition increases and then decreases. The best accuracy is obtained at λ = 0.5. This indicates that the contributions of both branches are equally important in the learning process. The CNN branch is biased to focus on more significant discriminative features, while the vision transformer branch focuses on more regions of discriminative features than the CNN. When the weights of the two branches are equal, the model can adaptively filter among the features with high relevance to the pedestrians, and then capture the comprehensive and significant discriminative features of the pedestrians, allowing the model to achieve the best performance.

### Comparison with existing relevant person ReID approaches

The results of the comparison with the existing relevant methods are shown in Table 9. The methods in the first group are local feature approaches based on fixed regions. The methods in the second group are semantic-based local feature methods using additional auxiliary models. The third group of methods is based on a CNN-based attention mechanism method, and the fourth group is a method that introduces vision transformer (where ${\rm{TransReI}}{{\rm{D}}^{\rm{*}}}$ means without a sliding-window setting).

**Table 9:** Comparison with existing relevant person ReID methods, in which ‘–’ means they do not report the corresponding results. The best performance is shown in bold.

	Methods	Market-1501		MSMT17	
		mAP (%)	Rank-1 (%)	mAP (%)	Rank-1 (%)
Stripe-based	RGA-SC (Zhang et al., 2020)	88.1	95.8	–	–
	PCB+RPP (Sun et al., 2018)	81.6	93.8	40.4	68.2
	MGN (Wang et al., 2018)	86.9	95.7	52.1	76.9
Extra semantic based	GASM (He & Liu, 2020)	84.7	95.3	52.5	79.5
	SPReID (Kalayeh et al., 2018)	81.3	92.5	–	–
	AANet (Tay, Roy & Yap, 2019)	83.4	93.9	–	–
	${p^2}$-Net (Guo et al., 2019)	85.6	95.2	–	–
	HOReID (Wang et al., 2020)	84.9	94.2	–	–
General attention methods of CNN-based	IANet (Hou et al., 2019)	83.1	94.4	46.8	75.5
	SCSN (Chen et al., 2020)	88.5	95.7	58.5	83.8
	ABD-Net (Chen et al., 2019)	88.3	95.6	60.8	82.3
	BAT-net (Fang et al., 2019)	85.5	94.1	56.8	79.5
Vision Transformer-based	DAT (Zhang et al., 2021)	89.5	95.6	61.2	82.3
	${\rm{TransReI}}{{\rm{D}}^{\rm{*}}}$ (He et al., 2021)	88.2	95.0	64.9	83.3
	PAT (Li et al., 2021)	88.0	95.4	–	–
	AAformer (Zhu et al., 2021)	87.7	95.4	62.2	83.1
	TCCNet (Ours)	**90.4**	**96.1**	**66.9**	**84.5**

**MSMT17.** On this dataset, our framework obtains the best performance, as shown in Table 9. And it outperforms all other methods by at least 4.7% and 0.8% in both mAP and Rank-1 identification rate, respectively. The compared methods are divided into three groups, *i.e*., local feature extraction methods based on fixed regions, methods using additional auxiliary models to extract semantic-level local features, and methods using vision transformer-based methods, and TCCNet outperforms all of these methods by a considerable advantage. MSMT17 is a large-scale dataset that covers a long period of time and presents complex illumination variations. This validates that our proposed framework can extract to the effective semantic information as well as locally significant information to retrieve difficult samples.

**Market1501.** Table 9 shows the evaluation of the Market1501 dataset. We compare the recent CNN-based and Transformer-based approaches with our model. From the results, we can see that our framework also achieves the best performance in Rank-1 identification rate and mAP. Since the performance of this dataset is close to saturation, the results of these state-of-the-art methods are very close.

## Conclusions

In this work, we proposed a novel end-to-end neural network, TCCNet, taking advantage of global self-attention mechanism and local convolution operator to capture features for person re-identification. Our framework was not only able to improve the ability to capture the structural diversity of the person features but also did well in capturing the relationship between local and global features to weaken the background clutter. Moreover, LFCM and HFCM were proposed for enhancing the complementarity of heterogeneous features to learn more comprehensive and salient features of the individual. The experiments demonstrated that TCCNet achieved competitive performance with mAP of 66.9% and Rank-1 identification rate of 84.5% on the MSMT17 dataset. The effectiveness of the model is also validated on the Market-1501 benchmark datasets. From the results of the visualization, TCCNet provides more precise coverage of the entire body area. Meanwhile, it enhances the focus on local distinguished areas without any additional pose estimation model.

In this article, the proposed method is purely for academic research in the publicly available datasets and the objects in the video are also blurred in the experiments, which does not cause the privacy concerns. Naturally, the privacy concerns are rising as the methods in video surveillance are utilized to practical applications. In the future, we will continue investigate how to conceal some of the private information given in photographs by studying on the principles of visual cryptography, signal mixing, and picture disruption to secure users’ privacy on person templates is required to satisfy public privacy concerns.

Although accuracy is the primary issue for specific Re-ID network architecture design, efficiency is also a significant consideration in Re-ID architecture design. Therefore, investigating how to use a framework or algorithm to improve performance while ensuring the computational efficiency of the model is an important issue as the field moves towards practical applications. In the future, we intend to further investigate the proposed model and explore more reasonable architectures to extract more effective local feature representations achieving the balance between accuracy and time.

## Additional Information and Declarations

### Data Availability

The following information was supplied regarding data availability: The code for this work is available at GitHub: https://github.com/LiYanchao-lab/TCCNet. The MSMT17 dataset is available at: https://www.pkuvmc.com/dataset.html. The Market1501 dataset is available at: https://zheng-lab.cecs.anu.edu.au/Project/project_reid.html.
